# Does Prior Training Affect Acute O_2_ Supply Responses During Exercise in Desaturator COPD Patients?

**DOI:** 10.2174/1874306400802010029

**Published:** 2008-03-13

**Authors:** Delphine Delample, Meritxell Sabate, Christian Préfaut, Fabienne Durand

**Affiliations:** 1 INSERM ERI25 «Muscle and Pathologies», F-34295 Montpellier, France. Université Montpellier I, EA4202, F-34295 Montpellier, France; 2 Laboratoire SSA “Sport Santé Altitude”, Département STAPS, Font-Romeu, France

**Keywords:** COPD, training program, oxygen, exercise.

## Abstract

**Background:**

This study investigated the effects of a prior individualized training program (TP) on the response to acute oxygen supply during exercise in chronic obstructive pulmonary disease (COPD) patients showing exercise-induced desaturation.

**Methods:**

Twenty-two COPD patients (mean [SD] FEV1 = 52.1 [3]% predicted) who desaturated on exercise participated in a TP. Exercise tolerance while breathing compressed air or oxygen was assessed using a walking test (WT) before and after TP. Oxygen flow was individualized.

**Results:**

Before TP, acute oxygen supply improved mean exercise tolerance. But this response was heterogeneous as only 8 patients increased their walking distance with oxygen. TP improved exercise tolerance in the entire population. However, a greater affect of oxygen administration during exercise was not observed after TP. The response to oxygen again showed great disparity as only 6 patients increased their walking distance with oxygen after TP.

**Conclusion:**

The response to oxygen supply during exercise varied among COPD patients. Moreover, despite the clinical benefits of TP, no cumulative effect of TP and oxygen supply was observed during exercise performance.

## INTRODUCTION

Exercise-induced desaturation is well reported in patients with chronic obstructive pulmonary disease (COPD) [1]. Indeed, some COPD patients do not exhibit clinical resting hypoxemia, i.e. an arterial oxygen pressure (PaO_2_) less than 55 mmHg, but they do show significant exercise-induced desaturation; these patients have been termed “desaturators” [2]. The six-minute walking test (WT) [1], a reliable clinical test to explore exercise tolerance in COPD patients, easily detects this desaturation. To correct this desaturation, oxygen is prescribed for desaturator patients to adjust the O_2_ saturation (SpO_2_) above 90% during exercise and especially the exercise included in rehabilitation programs. Recently, Bradley *et al*. [3] reviewed the evidence from single-assessment studies that this ambulatory O_2_ improves exercise performance in COPD patients but noted that the magnitude of improvement is unclear. The reports on the short-term O_2_ benefits to these patients during exertion are indeed conflicting; some of the studies mentioned in Bradley’s review reported an increase in exercise performance [4-9], whereas others did not [10, 11]. Moreover, the values of the variables investigated to assess the responses to O_2_ show great heterogeneity, which could lead to an average benefit that lacks significance. In fact, it may be that in some COPD patients O_2_ is available to the exercising muscles (because SpO_2_ is corrected) but is not used, suggesting that the peripheral muscles of these patients are unsuitable for O_2 _use.

Differences in exercise testing protocols, the prescribed flow, and/or disease severity have probably contributed to these divergent results [3]. For example, all the studies that reported a benefit from O_2_ used an O_2_ flow that was not individualized in relation to the O_2_ desaturation but was instead predetermined. As we know that the O_2 _supply has a dose-response effect [9], this methodology seems not well adapted and may be far from rehabilitation reality. Moreover, in all the studies that examined the short-term benefits of O_2 _in COPD, no data were given on the training status of the desaturator subjects. It has been suggested that the exercise-induced desaturation observed during a WT could be linked to muscle characteristics [1]. It is also well known that training can act on these muscle characteristics to improve muscle function [12], with not only increases in aerobic enzymes and the capillary density of leg muscles [13], but also a changed breathing pattern with higher tidal volume and lower breathing frequency [14, 15]. As a consequence, after TP, utilization of oxygen by COPD muscle should be more efficient and exercise performance increased.

This study thus tests the hypothesis that prior individualized training in desaturator COPD patients will increase acute O_2_ supply effects on the exercise performance.

## MATERIAL AND METHODS

### Population

Twenty-two patients with mild to severe COPD as defined by the Global Initiative for Chronic Obstructive Lung Disease guidelines [16] were recruited from an inpatient clinic before TP. All were desaturators as they presented significant O_2_ desaturation during WT, i.e. a fall in SpO_2_ of at least 4% over 3 minutes [1]. In addition, SpO_2_ had to be lower than 90% to be qualified for acute O_2_ supply, i.e. ambulatory oxygen therapy. All patients were stable at the time of the study and no one was treated by long-term oxygen therapy or suffered from clinical cardiovascular, neurological, or any other disease that might have contributed to dyspnoea or exercise limitation. The protocol was explained to each subject and all gave written informed consent.

### Initial Assessments

All patients underwent pulmonary function testing including spirometry and lung volumes determined by plethysmography (V6200 Autobox; Sensormedics; Yorba Linda, CA, USA). Measurements of resting arterial blood gas levels while the patients breathed room air were obtained during a period of clinical stability and quantified with a blood gas analyser (pHOx Plus L, Nova Biomedical, Waltham, MA, USA).

All patients performed a cardiopulmonary exercise test (CPET) on an electrically-braked cycle ergometer (Ergometrics 900; Ergoline; Bitz, Germany) following the individualized protocol usually used in our laboratory. On-line calculations of whole-body oxygen consumption (VO_2_) were averaged sequentially over 15-s intervals and displayed on a screen monitor to observe the progress of the test. Heart rate (HR) was measured continuously throughout the test by 12-lead electrocardiography. The ventilatory threshold was determined using the ventilatory equivalents of O_2_ and CO_2_ method [17].

### 6-Minute Walking Test (WT)

The WT was performed around a perimeter of 31.5 m. Each subject performed two tests to allow the observation of any variability due to “learning”, with a rest of approximately 1 hour between tests. The second test was performed only if SpO_2_ and HR were identical to the values before the first WT. Patients were asked to walk at their own maximal rate and we retained the greater distance of the two tests. They were asked to cover as much ground as possible in 6 minutes while maintaining a steady rate without running. No encouragement was given, and they were informed each minute of the remaining time. The patients were allowed to stop, but they could start again, if possible, within the allocated 6 minutes. Arterial oxygen saturation (SpO_2_) and HR were monitored throughout the walk every minute using a pulse oximeter (Nonin 8500 M; Nonin Medical, Inc., Minneapolis, MN, USA). The patients were asked to indicate their level of breathlessness using a visual analogue scale before and immediately after each walk.

### Determination of O_2_ Flow

As there is no official consensus to individualize O_2_ flow supply according to O_2_ desaturation during the WT, we used the methodology of our clinic. This methodology has been used for several years and provides good re-saturation with SpO_2_ equal to or above 90%. When the patient exhibits SpO_2_ between 90 and 85%, an O_2_ flow of 2 litres is given. When 84 ≥ SpO_2_ ≥ 80%, O_2_ flow amounts to 4 litres. For SpO_2_ < 80%, O_2_ flow is 5 litres. O_2 _flow was *via *cylinder and nasal specs.

### Training Program (TP)

All patients participated in an in-patient training program for 4 weeks. The whole-body exercise training included stationary cycling, walking, swimming, and team sports. The exercise intensity was individualized according to target heart rate, i.e. the heart rate at the ventilatory threshold (VT) determined during CPET. This intensity was continuously monitored with a cardiofrequency meter and maintained throughout all exercise. The desaturator COPD patients did not always exhibit O_2_ desaturation during TP and ambulatory O_2_ was only used when necessary. For most of the program, in fact, they did not receive O_2_. Training sessions were held 6 days per week and lasted 45 minutes on average. Training volume per week was 15.1 ± 1.2 h on average and divided in four sessions of stationary cycling (45 minutes per session), three sessions of swimming and team sports (45 minutes per session) and 4 sessions of walking (one or two hours per session). All sessions were supervised by a physiotherapist.

### Protocol

Initial assessments were made on admission to the clinic. One to three days after CPET, all patients performed 2 WTs on the same day. The first test (basal WT [BWT]) was performed while they breathed room air. The second test (AIR-WT) was performed while they breathed compressed air. At least one day later the patients were tested during WT whilst breathing oxygen (O_2_-WT). The O2-WT was performed twice as it is well known that the best performance is generally obtained from the second test. Oxygen flow was adjusted in all patients to reach an SpO_2_ > 90%. Before each WT, they breathed the gas mixture for 15 minutes. Two indistinguishable cylinders located at the middle of the walking perimeter, one with compressed air and one with O_2_, were connected to a 15-m tube ending in a nasal cannula. The patients did not know which gas was added. This experimental protocol was conducted by the same investigator before and after TP with the same flow of O_2_ and compressed air.

### Statistical Analysis

Results are expressed as mean ± SEM. For each variable of interest, a two-way analysis of variance (ANOVA) with repeated measures was performed. One way was assigned for training condition (before/after) and the other for O_2_ supply (without/with). When ANOVA detected a significant main effect, post-hoc comparisons were made using a Tukey test. The comparison between the oxygen responder group and the non-responder group was performed using an *unpaired t test*. Significant differences were declared when p<0.05.

## RESULTS

### Baseline Characteristics

Table **1** shows the baseline characteristics of all the patients. The patients present a marked limitation of their aerobic capacity (VO2max = 50.4 ± 3.4% predicted). They had moderate chronic obstruction (FEV_1_ = 52.1 ± 3% predicted) and mild resting hypoxemia (PaO_2_ = 67.4 ± 1.5 mmHg). All patients showed clinical desaturation with a mean fall in SpO_2 _equal to 12.1 ± 1.3% and SpO_2_ at the end of WT equal to 81.1 ± 1.5%. During the O_2_-WT, 6 patients required a flow of 2 L, 8 patients required 4 L, and 8 patients required 5 L. O_2_ administration thus prevented critical desaturation in all subjects and no patient had their SpO_2_ level fall to < 90% (End O_2_-WT SaO_2_ = 90.9 ± 0.6%).

### O_2 _Responses Before TP

**Fig.(1):**
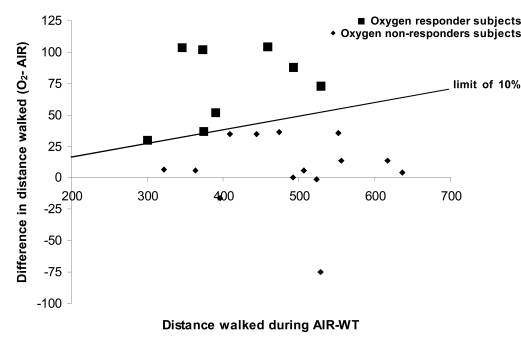
Individual data on walk distance on six minute walk test (WT) after breathing compressed air or oxygen.

Comparison of the distance walked during AIR-WT versus O_2_-WT showed a statistically significant effect of O_2_ on walking distance (Table **2**, p<0.05) with no change in HR at the end of WT. This increase in walking distance was associated with a significant effect of O_2_ on dyspnoea (p<0.05), which was 6% lower at the end of O_2_-WT than at the end of AIR-WT. No effect of O_2_ on HR at rest or at the end of WT was observed (Table **2**). Nevertheless, high variability was noted in the O_2_ response. In line with the literature, we considered the patients who increased their walking distance by at least 10% while breathing oxygen to be oxygen responders (see *Methodological considerations *in the discussion). Thus, compared with AIR-WT, during O_2_-WT 14 patients showed no improvement in exercise tolerance and 8 patients increased their walking distance (18.2 ± 2.7%, i.e. 74 ± 10.8 m on average) (Fig. **1**). No basal parameter predicted the occurrence of O_2 _benefits during O_2_-WT. However, oxygen responders seemed to have greater disease severity than non-responders (FEV_1_ = 46.8 ± 4.9% predicted *vs* 53.7 ± 3.5% predicted, respectively) as well as lower exercise tolerance (distance walked during AIR-WT before TP: 407.5 ± 27.5 m *vs* 487.2 ± 24.6% predicted, respectively; p<0.05).

### Effects of TP

Endurance capacity, measured as an increase in the distance walked during WT, improved significantly after 4 weeks of training (458.2 ± 20 m *vs* 512.4 ± 20.3 m; p<0.05) for the entire population. The mean gain was 13.2% after training, or 54.5 ± 12.9 m. Dyspnoea at rest and at the end of WT was significantly decreased after TP (p<0.05). TP had no significant effect on HR at rest or at the end of WT (Table **2**). The benefits of TP on exercise tolerance were the same in oxygen responders and non-responders (distance walked by oxygen responders increased by 67.4 ± 29.5 m and by non-responders, 46.6 ± 12.2 m).

### O_2_ Responses After TP

After TP, the same O_2_ flow allocated before TP was used. On average, O_2_ inhalation during WT increased the distance walked statistically but not clinically, compared with AIR-WT (512.4 ± 20.3 m *vs* 538 ± 19.9 m, respectively; p<0.05). O_2_ significantly decreased dyspnoea at the end of O_2_-WT (-9.3% between dyspnoea at the end of AIR-WT and dyspnoea at the end of O_2_-WT; p<0.05). Despite the greater distances covered, HR was not modified by acute oxygen supply, before or after WT.

Individual responses to O_2_ supply varied greatly. After TP, when patients breathed O_2_ during WT, 6 increased their walking distance by > 10% (18.7%, i.e. 73.1 m on average) but 16 showed no improvement. When we compared data before and after TP, only 4 patients who were able to increase their walking distance with O_2_ before TP had also improved their walking distance after TP. Thus, 4 patients who were oxygen responders before TP became non-responders after TP. And 2 patients who were oxygen non-responders before TP became responders after TP. Again, no baseline characteristic allowed us to predict either situation.

### Effect of Compressed Air on Walking Distance

Compressed air administration did not modify the results of the basal test in any patient. Walking distance during BWT was 415.4 ± 27 m and 419.5 ± 24.3 m, which corresponds to a 1.5% difference.

## DISCUSSION

The main result of this study reported that, despite the clinical benefits of TP, a prior TP did not optimize the acute effect of O_2_ supply on desaturator COPD patients during a walking test. We also confirmed that the responses to O_2_ supply of COPD patients show wide disparity during exercise.

### Methodological Considerations

#### Choice of exercise.

The 6-minute walking test has been validated for the detection of therapeutic improvements in exercise tolerance and dyspnoea. It thus has been widely used to show that O_2_ supply during exercise can improve tolerance [4, 5, 18]. Walking was reported to be associated with the lowest mean SpO_2_ value when saturation was measured during several daytime activities [19]. In addition, WT is more sensitive to desaturation than other tests like cardiopulmonary exercise testing on cycle ergometer [1], and it provokes breathlessness. It thus appears to be a functional test well adapted to evaluating patients in their own daily surroundings.

#### Protocol for O_2_ supply.

Contrary to other studies which gave a pre-determined FIO_2_ to their patients during exercise, we individualized the O_2_ flow on the basis of the desaturation observed at the end of AIR-WT. This ensured that each patient reached a SpO_2_ ≥ 90%. The O_2_ supply protocol described above is regularly used in our institution and personal observation confirms that this individualized protocol effectively prevents desaturation during exertion. In our study, all our patients kept their SpO_2 _≥ 90% during WT (SpO_2_ = 90.9 ± 0.7% at the end O_2_-WT).

#### Oxygen response.

Studies on O_2_ supply and its benefits always present their results as averages. A few recent studies have focused on the heterogeneity of these results. However, none have determined the threshold above which the increase in walking distance thanks to O_2_ supply is clinically significant. In the literature, the variability between two WTs is less than 10% in many chronic diseases [20-24]. Moreover, Eiser *et al*. [25] observed an 8% variability between the WTs of COPD patients. We thus assumed that a variation above 10% should not be considered as variability but as clinically significant modification.

### Results Discussion

Our study demonstrated that before TP O_2 _inhalation significantly increased the exercise tolerance of desaturator COPD patients. Several studies [5, 8, 26] have described improvements in the dyspnoea index and exercise performance when O_2_ was administered only during exercise. Some [8, 26] also noted difficulties in predicting the response to O_2_. In our study, no basal or WT parameter (especially exercise-induced desaturation) reliably predicted the response to O_2_ supply during exercise. Nevertheless, disease severity and exercise capacity before TP tended to be lower in the oxygen responders. On average, our patients presented moderate chronic airway limitation (FEV_1_ = 52.1 ± 3% predicted) and Fujimoto *et al*. showed that patients with moderate severity (FEV1 > 50% predicted) did not improve exercise capacity to the same extent as did patients with more severe disease, indicating that O_2_ supply during exercise might be useful only in patients with moderate to severe disease. Somfay *et al*. [9] demonstrated that O_2_ supply during exercise induced dose-dependent improvement in endurance and symptom perception in COPD patients. In our study, we used a low O_2_ flow, which was nevertheless individualized. It is possible that in these conditions, the response to O_2_ during exercise is less remarkable and not so clinically significant. But in our case, we observed the real effects of acute O_2 _supplementation as it is prescribed in medical institutions.

Most studies have reported a wide range of individual responses to O_2_, as did ours. It must be emphasized that the effects of hyperoxia are multifactorial and involve many integrated mechanisms. During O_2_ breathing in this study, the end O_2_-WT heart rate was similar to air-WT despite the greater distances covered by the patients. In addition, acute O_2_ supply significantly reduced perceived dyspnoea at the end of WT. Jolly *et al*. [7] suggested that cardiovascular limitation could be implicated in the response to O_2_ during exercise. Moreover, in addition to the diffusion limitation in the lung, a diffusion limitation at the level of the skeletal muscles may be present [27]. Supplemental O_2_ may thus fail to enhance muscle O_2_ utilization during exercise in COPD patients with peripheral muscle weakness [28]. One hypothesis to explain our heterogeneous responses is thus that in hyperoxic conditions, the above-mentioned mechanisms are involved but to different degrees in each patient.

On average, the response to O_2_ supply during exercise was not modified by TP. Although we observed an effect of O_2_ after TP on walking distance and dyspnoea, the combination of prior TP and acute O_2 _supply during exercise had no cumulative effect on the exercise tolerance of COPD patients who present exercise-induced desaturation. TP was, however, efficient in increasing exercise tolerance. Indeed, after training, the distance walked during WT was statistically and clinically increased and dyspnoea was significantly decreased with a heart rate at the end of the WT similar to that before TP. These results confirm the beneficial effects of individualized training on exercise tolerance in COPD patients. Exercise training induces cardiovascular benefits (with a gain in spontaneous baroreflex sensitivity) [29] and pulmonary benefits. Casaburi *et al*. [12] demonstrated more efficient exercise breathing patterns and lower ventilation (with increase in tidal volume). TP also produces changes in skeletal muscles (increased oxidative metabolism activity and mean fibre area) [13, 30]. In summary, TP improves exercise tolerance by inducing central (cardiopulmonary) and peripheral (peripheral skeletal muscle) adaptations. The success of O_2 _supply is also likely to depend on its net effects on cardiopulmonary and peripheral muscle functions [31]. As no cumulative effect of TP plus acute O_2_ supplementation was seen, we can hypothesize that the training modalities of our TP led to central adaptations but could not be adjusted enough to induce peripheral adaptations. More resistance training in this TP would probably induce peripheral adaptations more efficiently since little demand is placed on the cardiopulmonary system [32] during this type of exercise, making it well tolerated by COPD patients. But possibly we did not observe a greater response to O_2_ after TP because the effects of TP far exceeded the acute effects of O_2_. Many factors account for the absence of a cumulative effect of training and O_2_ supply during exercise. The contribution of exercise-induced desaturation to exercise limitation is still uncertain, and the mechanisms by which O_2_ affects exercise tolerance are complex. The present study confirmed previous reports that showed that the acute effects of O_2_, as well as the effects of training, do not correlate with lung function parameters or blood gas values at rest or during exercise [4, 33, 34]. Furthermore, in the present study, the acute effects of O_2_ did not predict the effects of training in individual subjects. Hence, patients with a poor response to O_2_ are not necessarily poor candidates for training. These findings suggest that oxygen and training may influence exercise tolerance by different mechanisms.

In summary, this study confirmed the great heterogeneity in responses to O_2_ supply during exercise, with approximately 35% of our COPD patients improving their exercise tolerance. The training program did not modify this response to O_2_, suggesting that efficient O_2_ utilization does not depend on muscular and central benefits of TP. Moreover, basal parameters and exercise-desaturation do not seem to be reliable criteria for selecting those patients who will most benefit from O_2_ during activities. As the response to this therapy is unpredictable, every patient should be individually evaluated.

## Figures and Tables

**Table 1.** Anthropometric, Spirometric and Physiological Data of COPD Patients Before Training Program

Variables	Values
Age (yr)	61.9 ± 1.9
Female: Male	07: 15
BMI (kg/m^2^)	26.1 ± 0.9
Peak VO_2_ (%predicted)	50.4 ± 3.4
Maximal HR (bpm)	125 ± 7
HR at ventilatory threshold (bpm)	115 ± 6
FEV_1_ (% predicted)	52.1 ± 3
FEV_1_/FVC (%)	55.7 ± 2.7
PaO_2_ (mmHg)	67.4 ± 1.5
AIR-WT distance (m)	458.2 ± 20
Baseline dyspnoea	1.3 ± 0.3
End AIR-WT dyspnoea	6.1 ± 0.6

*Definition of abbreviations:* BMI = body mass index; FVC: forced vital capacity; HR: heart rate. Values are mean ± SEM.

**Table 2:** Effects of Acute Oxygen Supplementation and Training Program (TP) on WT in COPD Patients

Variables	Before TP		After TP	
	AIR	O_2_	AIR	O_2_
Distance (m)	458.2 ± 20	489.6 ± 19.6†	512.4 ± 20.3*	537.9 ± 19.9*†
SpO2 at rest (%)	93.2 ± 0.6	96.9 ± 0.4†	95 ± 0.8	97,1 ± 0.5†
SpO2 at the end of the test (%)	81.1 ± 1.5	90.9 ± 0.7†	81.1 ± 1.4	91.3 ± 1†
Dyspnoea at rest	1.3 ± 0.3	0.9 ± 0.3	0.7 ± 0.3*	0.8 ± 0.3*
Dyspnoea at the end of the test	6.1 ± 0.6	5.5 ± 0.6†	5.6 ± 0.7*	4.7 ± 0.6*†
HR at rest (bpm)	92 ± 2.8	90.4 ± 2.5	89.8 ± 2.7	90.6 ± 2.8
HR at the end of the test (bpm)	117.7 ± 3	115.1 ± 3.4	119.3 ± 2.7	117 ± 3.4

Values are represented as mean ± SD.

* : p<0.05 within group comparison before versus after rehabilitation.

† : p<0.05 comparison between O_2_-WT and the corresponding AIR-WT.
